# Surgical management of tuberculous constrictive pericarditis in a developing country: case report

**DOI:** 10.1186/1749-8090-10-S1-A186

**Published:** 2015-12-16

**Authors:** Sotheenathan Krishinan, Basheer Ahamed Abdul Kareem, Abu Yamin Khamis, Ahmadi Salleh, Sivasangari Subramaniam, Mohd Hamzah Kamrulzaman

**Affiliations:** 1 Department of Cardiothoracic, Penang General Hospital, Georgetown, Penang, 10990, Malaysia; 2 Clinical Research Center, Penang General Hospital, Georgetown, Penang, 10990, Malaysia

## Background/Introduction

Tuberculosis has been increasing in incidence in recent years especially in developing countries. Tuberculous pericarditis is a form of extra pulmonary tuberculosis that is considered unusual.

## Aims/Objectives

We report a case of tuberculosis of pericardium complicated with constrictive pericarditis and the surgical challenges encountered.

## Method

Diagnosis is often challenging. High indexes of suspicion combined with the use of all available diagnostic techniques are important to increase diagnostic yield. A definite or proven diagnosis is based on demonstration of tubercle bacilli in pericardial fluid or on histologic section of the pericardium. Constrictive pericarditis is a complication of tuberculous pericarditis that necessitates surgical intervention.

## Results

The timing of surgical intervention is controversial, but many experts recommend a trial of medical therapy for non-calcific pericardial constriction, and pericardiectomy in non-responders after 4 to 8 weeks of anti-tuberculosis chemotherapy.

## Discussion/Conclusion

Pericardiectomy is a surgical option which carries high mortality and morbidity. We report a case of tuberculosis of pericardium complicated with constrictive pericarditis and the surgical challenges both intraoperative and post operatively.

